# *Idiomarina aminovorans* sp. nov., a halophilic bacterium isolated from the Llamara salt pan in the Atacama Desert of northern Chile

**DOI:** 10.1007/s00792-025-01399-x

**Published:** 2025-07-21

**Authors:** Christian Hintersatz, Luis Antonio Rojas, Sean Ting-Shyang Wei, Sabine Kutschke, Angela Thewes, Falk Lehmann, Rohan Jain, Katrin Pollmann

**Affiliations:** 1 Department of Biotechnology, Helmholtz-Institute Freiberg for Resource Technology, Helmholtz-Zentrum Dresden-Rossendorf, Bautzner Landstraße 400, 01328 Dresden, Germany; 2 Department of Chemistry, Universidad Católica del Norte, Av. Angamos 0610, 1270398 Antofagasta, Chile; 3 Department of Biogeochemistry, Institute of Resource Ecology, Helmholtz-Zentrum Dresden-Rossendorf, Bautzner Landstraße 400, 01328 Dresden, Germany

**Keywords:** Novel species, Halophilic bacteria, Isoprenoid biosynthesis, Extremophile, *Idiomarina*, Atacama Desert

## Abstract

The Atacama Desert, one of the most extreme environments on our planet, harbors a plethora of unique microbial communities adapted to the harsh conditions of the habitat. In this study, strain ATCH4^T^, a novel Gram-stain-negative, curved rod-shaped bacterium, was isolated from the Llamara salt pan, located in the Atacama Desert in the north of Chile. ATCH4^T^ was capable of growth within a range of 3–12% (w/v) NaCl, 4–40 °C, and pH 6–9. Comparative 16S rRNA analysis placed the strain within the genus *Idiomarina*, with its closest related type species being *I. loihiensis*, *I. ramblicola*, and *I. abyssalis*. Genomic analysis revealed the presence of several genes linked to halophilicity as well as unique metabolic pathways, including the ability to synthesize C5 and C10–C20 isoprenoids, which may contribute to the isolate’s survival in hypersaline conditions. The observed isoprenoid biosynthesis pathways suggest potential applications in various biotechnological fields, including the production of biofuels, pharmaceuticals, and other valuable chemicals. Levels of DNA-DNA relatedness, average nucleotide identity, and several phenotypic and chemotaxonomic markers clearly indicate that strain ATCH4^T^ represents a novel species of the genus *Idiomarina*, for which the designation *Idiomarina* *aminovorans* sp. nov. (type strain ATCH4^T^ = DSM 114475 = LMG 32710) is proposed.

**Supplementary Information:**

The online version contains supplementary material available at 10.1007/s00792-025-01399-x.

## Introduction

Despite its extreme environmental conditions, the Atacama Desert comprises numerous ecological niches, harboring a surprising microbial diversity. Specifically, the salt flats located in the northern parts of the desert represent a unique habitat. While the Atacama Desert is one of the most arid places on Earth, these saltpans are significantly more humid, allowing even genera typically connected to marine environments, such as *Idiomarina*, to thrive.

*Idiomarina*, the type genus of the family *Idiomarinaceae* within the order *Alteromonadales* of the class *Gammaproteobacteria*, as well as the corresponding type species *Idiomarina abyssalis,* were originally proposed by Ivanova et al. in 2000. The family consists of the three genera *Idiomarina*, *Pseudidiomarina* (Dar Jean et al. 2006), and *Aliidiomarina* (Huang et al. 2011). To date, the genus *Idiomarina* comprises 13 species with validly published names (https://lpsn.dsmz.de/genus/idiomarina). The vast majority of these species were isolated from marine (Brettar et al. 2003; Song et al. 2013) and hypersaline environments (Choi and Cho 2005; Martinez and Butler 2007; Yoon et al. 2007; Taborda et al. 2009; Zhong et al. 2014; Lee et al. 2015; León et al. 2015). Additionally, novel type strains have been isolated from more unconventional sources, such as fermented fish (Sitdhipol et al. 2013), the rhizosphere of a mangrove forest (Chen et al. 2019), or a hydrothermal vent (Donachie et al. 2003). Representatives of the genus *Idiomarina* are Gram-stain-negative, motile rods with a slightly curved shape that form non-pigmented or faint yellow colonies. They are strictly aerobic, moderately halophilic, and catalase- as well as oxidase-positive. For their growth, NaCl concentrations within the range of 0.5–20% (w/v) are required with optimal conditions between 1–10% (w/v). Q-8 is the predominant respiratory ubiquinone, and the major fatty acids are primarily iso-branched. The DNA G + C content ranges from 45 to 54 mol% (Ivanova et al. 2000; Taborda et al. 2009).

In this study, strain ATCH4^T^, a novel species of the genus *Idiomarina* was isolated from the Llamara salt pan located in the Atacama Desert of northern Chile and characterized utilizing a polyphasic approach. Strain ATCH4^T^ was discovered during a broader investigation aimed at isolating novel siderophore-producing microorganisms from hypersaline environments, the results of which have been previously published (Hintersatz et al. 2023). Although not the primary focus of that study, ATCH4^T^ was indicated to be taxonomically distinct and therefore subjected to further analysis. The characterization of such strains is important for obtaining insights into microbial diversity in extreme environments, which could reveal unique adaptations and contribute to our knowledge of biogeochemical cycles. Additionally, these microorganisms may serve as a source for novel compounds with potential applications in biotechnology and pharmaceuticals.

## Material and methods

### Isolation and cultivation

Strain ATCH4^T^ was isolated from surface waters of the Llamará salt pan (21°21′28″S, 69°35′56″W), which is located in the Atacama Desert in the north of Chile. The concentrations of typically occurring anions and cations present in the sample were determined using anion-chromatography and inductively coupled plasma—mass spectrometry. Based on these measurements, an isolation medium was designed to accommodate the strain’s growth conditions. The resulting medium designated IM4 (0.1 g/l LiCl, 0.8 g/l CaCl_2_ × 2 H_2_O, 8 g/l MgSO_4_ × 7 H_2_O, 130 g/l NaCl, 2.6 g/l K_2_SO_4_, 30 g/l Na_2_SO_4_, and 2.5 g/l casamino acids, pH 8 with 1 M NaOH) was utilized for the subsequent isolation of ATCH4^T^.

For this, a tenfold dilution series of the sample was created with sterile 13% (w/v) NaCl solution, of which 100 µl were plated on IM4 solidified with 1.5% (w/v) agar. After two weeks of growth, colonies were picked and further cultured in liquid medium. Subsequently, the strain was replated twice more to ensure purity. For long-term storage, the culture was supplemented with 30% (v/v) glycerol and stored at −80 °C.

### 16S rRNA sequencing and phylogenetic analysis

Genomic DNA of strain ATCH4^T^ was extracted and purified via Nukleospin DNA RapidLyse kit (Macherey–Nagel) following the manufacturer’s instructions. For initial investigations, the isolate’s 16S rRNA gene sequence was amplified via PCR utilizing the universal primers 7F (5′-AAGASTTTGATYNTGGCTCAG-3′) and 1513R (5′-TACGGYTACCTTGTTACGACTT-3′). A 25 µl PCR reaction contained 50 ng of genomic DNA, 200 µM dNTPs, 0.2 µM of each primer, 0.1 µl DreamTaq DNA polymerase, and 2.5 µl 10 × DreamTaq buffer. PCR conditions included an initial denaturation at 98 °C for 2 min, followed by 30 cycles of amplification (30 s denaturation at 98 °C, 30 s annealing at 53 °C, 90 s extension at 72 °C) and a final extension at 72 °C for 10 min. The obtained PCR product was purified utilizing the MSB® Spin PCRapace kit (Invitek) and sequenced via Sanger sequencing. The resulting 16S rRNA gene sequence was compared to reference strains deposited in the GenBank database using Nucleotide BLAST. Phylogenetic analysis of the sequence data was conducted in MEGA11 (Tamura et al. 2021). The multiple sequence alignment tool MUSCLE (Edgar 2004) was utilized to align the strain’s 16S rRNA sequence with reference sequences of closely related type species. Based on the resulting alignments, phylogenetic trees were inferred via Maximum likelihood, Neighbor-Joining, and Minimum-Evolution method employing the Kimura-2-parameter model (Kimura 1980). The tree topology’s confidence level was assessed by bootstrap resampling method based on 1000 replicons (Felsenstein 1985).

### Genomic analysis

The whole genome of strain ATCH4^T^ was obtained via HiSeq sequencer (250 bp, pair-end). Low-quality raw reads (quality score < 30) were trimmed employing Trimmomatic 0.40 (Bolger et al. 2014). The remaining reads were assembled in SPAdes 3.15.3 (Bankevich et al. 2012) using the genome of *I. loihiensis* L2-TR^T^ as reference to obtain a draft genome. Annotation of the draft genome was carried out via the NCBI Prokaryotic Genome Annotation Pipeline (Tatusova et al. 2016). The calculation of the genome’s general statistics, including the number of contigs, N50, and G + C content (%), was carried out in QUAST (Gurevich et al. 2013). ContEst16S (Lee et al. 2017) was utilized to extract and estimate contaminations within the genomes based on the presence of 16S rRNA fragments, while the completeness and contaminations of the draft genome itself were evaluated with CheckM (Parks et al. 2015) based on the presence and duplication of single-copy marker genes. Average Amino Acid Identity (AAI) analysis of isolate ATCH4^T^ was conducted employing the AAI-profiler provided by the Holm research group of the University of Helsinki (http://ekhidna2.biocenter.helsinki.fi) (Medlar et al. 2018). Further, Average Nucleotide Identity (ANI) and digital DNA-DNA hybridization (dDDH) values between strain ATCH4^T^ and closely related species were determined with the OrthoANIu algorithm (Yoon et al. 2017) provided by the EZBioCloud web service and the Genome-to-Genome Distance Calculator (GGDC 3.0) (Meier-Kolthoff et al. 2022), respectively. For this, the genomes of related species were obtained from the NCBI prokaryotic reference genome database.

Next, using the UBCG2 pipeline (Kim et al. 2021), 81 single-copy core genes from the novel strain’s and reference type strains’ genomes were extracted, aligned, and concatenated. The alignment was conducted nucleotide-based instead of codon-based, while other settings were set to default. The resulting concatenated alignment was used to infer a phylogenomic tree using RAxML (Stamatakis 2014). The robustness of the nodes was estimated from gene support indices (GSI) with the default value of 95.

Lastly, the metabolic pathways of strain ATCH4^T^ and closely related species, along with genes facilitating the halophilicity of the novel isolate were inferred from their genomes using the Kyoto Encyclopedia of Genes and Genomes (KEGG) database. The KEGG tool BlastKOALA (Kanehisa et al. 2016) was used for K number assignment, while the pathway reconstruction was carried out with KEGG Mapper (Kanehisa and Sato 2020).

### Phenotypic characterization

The morphology and motility of strain ATCH4^T^ in its exponential growth phase were observed by phase-contrast microscopy (BX43, Olympus) with cells grown for one day at 30°C in IM4. The presence and position of the cells’ flagella were investigated via Leifson staining method (Piccolomini et al. 1999). In order to determine if the isolate is capable of anaerobic growth, cells were grown on IM4, supplemented with 1 g/l NaNO_3_, and incubated for 7 days at 30°C in an anaerobic jar. The generation of an anaerobic atmosphere was achieved by means of the Anaerocult™ A system (Merck). Oxidase and catalase activities were tested according to the methods described by Smibert & Krieg (1994), while the strain’s ability to hydrolyze starch, urea, casein, or DNA enzymatically was determined based on the protocols described by Hansen & Sørheim (1991).

The strain’s growth ranges and optima were investigated in a complex medium containing 5 g/l peptone, 1 g/l yeast extract and 0.1 g/l ferric citrate. If not stated otherwise, the medium contained 7% (w/v) NaCl, the pH value was set to 8 with 1 M NaOH and incubation was carried out at 30°C. For the investigation of growth range and optimum in regard to salt, the medium was supplemented with various amounts of NaCl (0, 0.5, 1, 3, 5, 7, 10, 12, 15, 17, 20% (w/v)). To elucidate temperature preferences, liquid cultures were incubated at 4, 20, 30, 40, and 50°C. To determine ATCH4^T^'s pH growth range and optimum, the medium was supplemented with 0.1 M MES (pH 5.5–6.5), HEPES (pH 7–8), or CAPSO (pH 8.5–10). The evaluation of the experiments was done based on the OD_600_ measured after one day of growth.

Utilization of a variety of substrates as sole carbon sources was investigated with Biolog Gen III plates using a suspension medium containing 2 g/l NH_4_Cl, 2 g/l MgSO_4_ × 7 H_2_O, 70 g/l NaCl, and 0.01% tetrazolium chloride. The pH of the suspension medium was adjusted to 7.5 using 1 M NaOH. The results were assessed based on the development of purple discoloration due to formazan formation after 7 days of incubation at 30°C. Further biochemical properties of both strains were determined using the API 20NE and API ZYM test kits (bioMérieux) according to the manufacturer’s instructions. One exception is the utilization of a 7% (w/v) saline solution for the preparation of cell suspensions and the supplementation of the AUX medium provided in the API 20NE test kit with 7% (w/v) NaCl.

### Chemotaxonomic analysis

For the chemotaxonomic analysis of strain ATCH4^T^, cells were grown on marine agar (Difco) for 24 h at 30°C. After harvesting, the cells were washed with sterile 7% NaCl (w/v) and subsequently lyophilized for later analysis. Respiratory quinones and fatty acid composition of the strain were determined by the identification service of the German Collection of Microorganisms and Cell Cultures (DSMZ) (Braunschweig, Germany).

## Results and discussion

### 16S rRNA sequencing and phylogenetic analysis

The 16S rRNA gene sequence of strain ATCH4^T^ was 1412 bp long. BLAST analysis unambiguously placed the isolate within the genus *Idiomarina*. Based on 16S rRNA sequence similarity, the species closest related to ATCH4^T^ are *Idiomarina loihiensis* (99.1%), *Idiomarina ramblicola* (98.7%), and *Idiomarina abyssalis* (98.1%)*.* The strain’s 16S rRNA sequence was deposited at GenBank under the accession number OM536012.

**Fig. 1:**
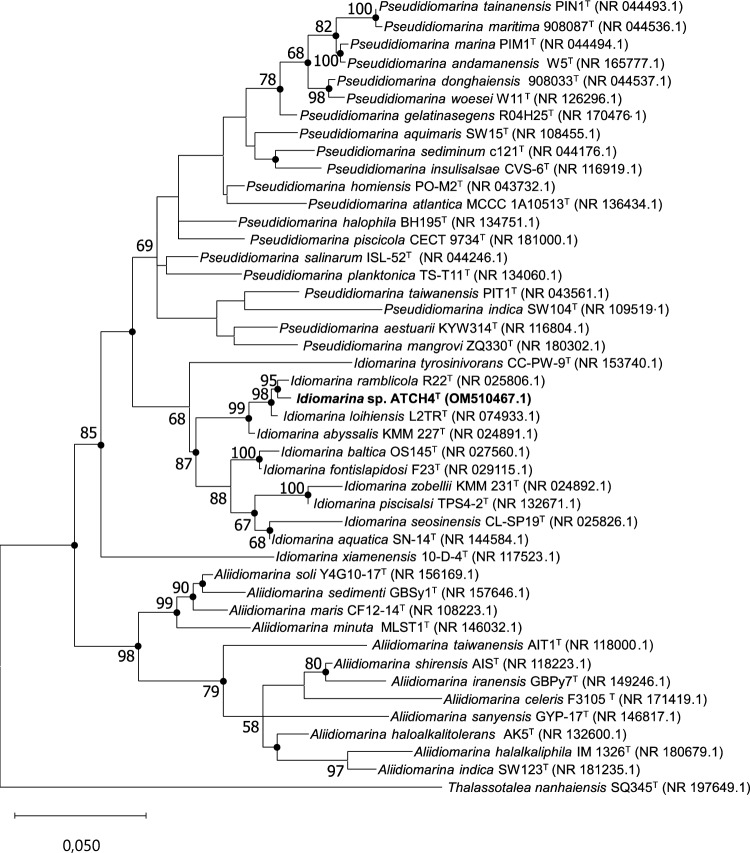
Maximum likelihood phylogenetic tree based on 16S rRNA gene sequences of strain ATCH4^T^ and related species belonging to the family *Idiomarinaceae*. Filled circles indicate branches recovered by maximum likelihood, neighbor-joining, as well as minimum-evolution methods. *Thalassotalea nanhaiensis* SQ354^T^ was used as outgroup. Only bootstrap values greater than 50% are given at the branch points. The scale bar represents substitutions per nucleotide position

Figure 1 depicts the maximum likelihood phylogenetic tree inferred from the alignment of ATCH4^T^'s 16S rRNA gene sequence with those of closely related type species.

Within all reconstructed phylogenetic trees, strain ATCH4^T^ forms a stable clade with the type strains of *I.* *abyssalis*, *I.* *loihiensis*, and *I. ramblicola*. Although the 16S rRNA gene sequence similarity was found to be higher between ATCH4^T^ and *I. loihiensis* (99.1%), the phylogenetic tree indicates a closer evolutionary relationship between the novel isolate and *I. ramblicola*. The sister genera *Pseudidiomarina* and *Aliidiomarina* form clearly distinguished clades from isolate ATCH4^T^, further supporting its position within the genus *Idiomarina*.

Due to their close genetic relationships, the type strains of the species within the same clade as ATCH4^T^ were employed as reference strains for subsequent phenotypic characterization.

### Genomic analysis

The whole genome sequencing of strain ATCH4^T^ via HiSeq sequencer yielded a total of 789,939 reads. The assembled draft genome is composed of 61 contigs with a total length of 2,752,493 bp and has 100% completeness without contamination. The sequencing coverage is 138 × and the N50 is located at 418,067 bp. The genome has a G + C content of 46.55 mol%, thus lying within the typical range reported for the genus *Idiomarina* (45–54 mol %). According to the analysis conducted with ContEst16S, no 16S rRNA gene sequence contamination was present. The draft genome of strain ATCH4^T^ has been deposited in the GenBank database under the accessions JAKNDA000000000.

**Fig. 2:**
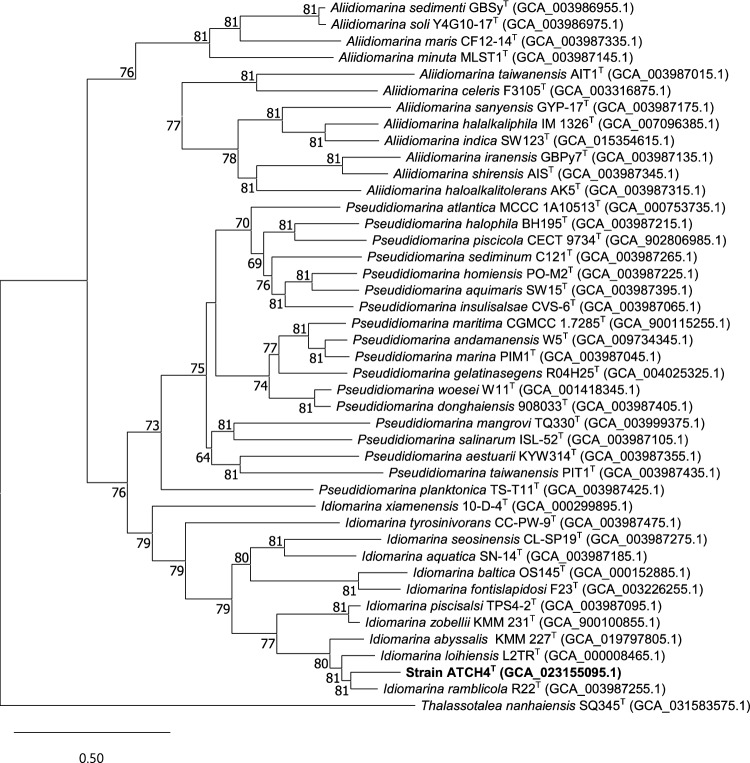
Maximum likelihood phylogenomic tree inferred on the basis of 81 single-copy marker genes found in the genomes of strain ATCH4^T^ and the type strains of closely related species. *Thalassotalea nanhaiensis* SQ354^T^ was used as outgroup. Gene support indices > 60 are give at the branch points. Scale bar represents substitutions per nucleotide position

The phylogenomic tree inferred on the basis of 81 core genes (Fig. 2) further supports the evolutionary relationships indicated by phylogenetic analysis.

The three genera of the family *Idiomarinaceae* form clearly distinguished branches within the phylogenomic tree. Consistent with the 16S rRNA phylogeny, strain ATCH4^T^ is positioned within a stable clade together with *I. abyssalis*, *I. loihiensis*, and *I. ramblicola*. Notably, ATCH4^T^ forms a distinct and well-supported lineage within this clade, as indicated by high gene support indices at the relevant nodes.

**Fig. 3:**
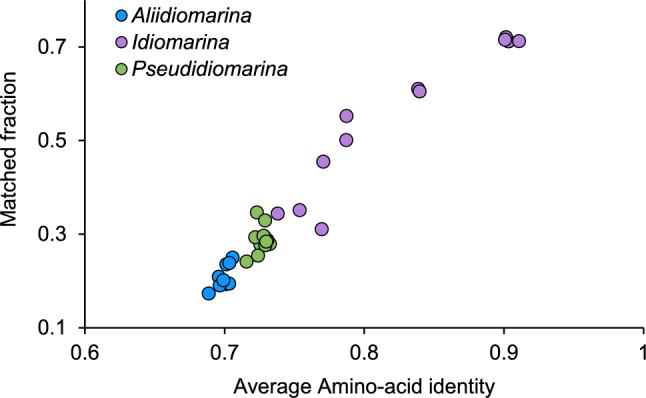
Average amino acid identity and fraction of query proteins with a match in the Uniprot database. Each data point represents a species

To further confirm the classification of strain ATCH4^T^ as a novel species, a suite of overall genome relatedness indices were employed. First, average amino acid identity (AAI) analysis was performed, providing robust evidence that ATCH4^T^ clusters within the genus *Idiomarina* (Fig. 3).

The high homology coverage (matched fraction: 0.31–0.72) and AAI values (0.75–0.91) between isolate ATCH4^T^ and members of *Idiomarina* strongly supports its placement within this genus. These values greatly exceed the generally accepted threshold for genus delineation, which are 0.45–0.65 for AAI (Konstantinidis et al. 2017). In contrast, comparisons with its sister genera *Aliidiomarina* and *Pseudidiomarina* yielded lower matched fractions (0.17–0.35) and lower AAI values of 0.17–0.25 and 0.72–0.73, respectively. These results are consistent with phylogenetic and phylogenomic analysis, which indicate that ATCH4^T^ is more closely related to *Pseudidiomarina* than to *Aliidiomarina*, but is most appropriately classified within *Idiomarina*.

Next, ANI and dDDH analysis was performed to clarify the species-level distinctiveness of strain ATCH4^T^. The calculated ANI and dDDH values calculated between the novel isolate and its most closely related type species are < 83.69% and < 26.6%, respectively (Table 1). These values are well below the generally accepted species boundaries of 96% (ANI) and 70% (dDDH) (Chun et al. 2018), providing strong genomic evidence that ATCH4^T^ represents a novel species within the genus *Idiomarina*.

**Table 1:** ANI and dDDH values calculated between the genomes of strain ATCH4^T^ and its five closest related type species

Reference genome	ANI [%]	DDH
*I. abyssalis*	82.02	23.9
*I. ramblicola*	83.69	26.6
*I. loihiensis*	81.99	24.2
*I. piscisalis*	75.28	18.3
*I. zobellii*	75.32	18.7

**Fig. 4:**
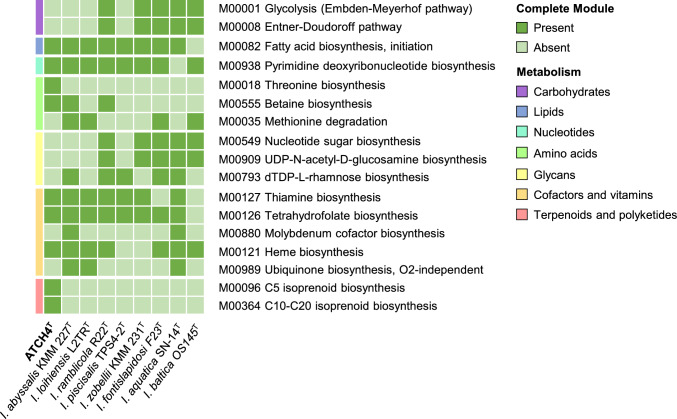
Comparative overview of the absence or presence of metabolic pathways distinguishing the novel strain ATCH4^T^ from its closest phylogenetic relatives

The analysis of complete and incomplete metabolic pathways in the genomes of strain ATCH4^T^, and its eight closest related type species via the KEGG tools BlastKOALA and KEGG mapper showed that key metabolisms, such as the core modules of glycolysis (M00002), gluconeogenesis (M00003), the citrate cycle (M00009), or the shikimate pathway (M00022) were present in all investigated strains (Table S1 in SI). However, the analysis also revealed an array of intergeneric variations that further distinguish the novel isolate from its phylogenetic relatives (Fig. 4).

Among its phylogenetic relatives, ATCH4^T^ is the only one capable of synthesizing threonine (M00018). This metabolic trait might provide the isolate with a competitive advantage in nutrient-limited conditions allowing it to thrive in environments with low threonine availability. The presence of this pathway indicates a unique ecological adaption from other *Idiomarina* species. Notably, ATCH4^T^ also exhibits a unique ability to synthesize C5 (M00096) and C10–20 (M00364) isoprenoids, a capability absent in its related species. C5 isoprenoids mainly function as building blocks for larger isoprenoids, such as hopanoids, terpenes, or other specialized secondary metabolites (Hunter 2011). These larger isoprenoids can be components of cell membranes, increasing their rigidity, while reducing permeability at the same time, especially in the presence of salts (Jordan et al. 2019). The ability to synthesize these compounds likely contributes to the strain’s resistance to the harsh conditions found in the Atacama Desert, including high salinity and extreme temperatures. Simultaneously, the capability of isoprenoid biosynthesis could position ATCH4^T^ as valuable organisms for biotechnological applications. Isoprenoids have a variety of commercial uses, including as pharmaceuticals, in fragrances, nutritional supplements, flavouring agents, or in advanced biofuels (Wang et al. 2021). Thus, the isoprenoids synthesized by the novel strain should be further investigated and characterized in future studies to assess their potential.

Furthermore, the analysis of the strain’s genome gave insights into the adaptation of ATCH4^T^ to its salt-rich native habitat. The halophilicity of the isolate is likely based on a combination of compatible solute and salt-in strategy. This assumption is supported by the presence of several genes involved in either the biosynthesis and transport of various osmolytes or potassium uptake. The KEGG orthology numbers of the recovered genes as well as the proteins are listed in Table S2. Identified genes, which are reported to be associated with the compatible solute strategy include *betABT* (glycine-betaine biosynthesis), *proABC* (proline biosynthesis), *gltBD* (glutamate biosynthesis), *glnA* (glutamine biosynthesis), *opuD* (osmolyte transport), *natAB* and *nhaC* (sodium efflux), as well as *mscS* (solute release) (Bashir et al. 2014; Remonsellez et al. 2018; Wang et al. 2023; Kaur and Kaur 2024). On the other hand, recovered genes, which are linked to the salt-in strategy are *trkAH* (potassium uptake) (Lee and Kim 2022).

### Phenotypic characterization

Cells of strain ATCH4^T^ were curved rods, 2–3 × 1.5–3.4 µm in size and Gram-stain-negative. They are strictly aerobic and motile by means of a polar flagellum. When grown on the isolation medium, the strain formed circular, convex, smooth, beige transparent colonies with irregular margins and 1–3 mm diameter. Growth was observed within a NaCl concentration range of 3–12% (w/v) (optimum 7–10% (w/v)), temperatures of 4–40°C (optimum 30°C), and pH levels between 6–9 (optimum 7). The NaCl tolerance range of ATCH4^T^ was distinct among the investigated species. Strain ATCH4^T^ tested positive for both oxidase and catalase activity, lacked the ability to reduce nitrate, but demonstrated the ability to produce H₂S. The strain exhibited hydrolysis of casein and gelatin, whereas aesculin hydrolysis was not observed. The isolate’s preferences for various substrates further distinguish it from related species (Table 2). The totality of properties determined for strain ATCH4^T^ is listed in the species description.

**Table 2:** Selected phenotypic properties differentiating strain ATCH4^T^ from type strains of closely related species within the genus *Idiomarina*; Strains: 1, ATCH4^T^ (data from this study); 2, *I. loihiensis* L2-TR^T^ (data from this study) 3, *I. abyssalis* KMM 227^T^ (Ivanova et al. 2000); 4 *I. ramblicola* R22^T^ (Martínez-Cánovas et al. 2004)

Characteristic	1	2	3	4
Cell shape	Curved rods	Slightly curved rods	Rods	Slightly curved rods
Cell size (µm)	2–3 × 1.5–3.4	0.7–1.8 × 0.35–0.45	1–1.8 × 0.7–0.9	2–3 × 0.75
Pigmentation	Beige	Beige to yellow	Non-pigmented	Cream
NaCl range (% w/v)	3–12	0.5–20	0.6–15	0.5–15
NaCl optimum (% w/v)	7–10	7.5–10	3–6	3–5
Temperature range (°C)	4–40	4–46	4–30	15–40
Temperature optimum (°C)	30	30	20–22	32
pH range	6–9	5.5–9.5	5.5–9.5	5–10
pH optimum	7.5–8	7–8	7.5–8	7–8
DNA G + C content (mol%)	46.6	47.4	47.2	48.7
Nitrate reduction	−	+	+	−
Production of H_2_S	+	−	−	+
Hydrolysis of:				
Casein	+	−	−	+
Aesculin	−	−	−	+
Gelatin	+	+	−	+
Utilization of:				
Maltose	−	+	−	−
Glycerol	−	+	+	−
Myo-inositol	−	+	ND	ND
D-glucose	−	+	−	ND
Acetic acid	+	+	+	−
Citric acid	−	+	−	−
Lactic acid	−	+	−	−
Propionic acid	+	+	+	−
L-aspartic acid	+	-	−	ND
L-glutamic acid	+	+	−	ND
L-alanine	+	+	+	−
L-serine	+	+	−	−
β-hydroxybutyric acid	+	−	−	+

+ positive, − negative, *ND* no data available

### Chemotaxonomic analysis

The major fatty acids of strain ATCH4^T^ are C_15:0_ iso (23.84%), C_17:0_ iso (17.69%), and C_17:0_ cyclo (11.5%) (Table 3). Among the evaluated strains, the high content of C_17:0_ cyclo fatty acid is unique to ATCH4^T^. In addition, it was the only strain for which C_19:0_ cyclo ω7c was detected, further distinguishing the isolate from its closest relatives. Cyclopropane fatty acids have been found to enhance bacterias’ resistance to osmotic stress, temperature fluctuations, and pressure by increasing membrane rigidity and reducing membrane permeability (Guillot et al. 2000; Chen and Gänzle 2016; Cronan and Luk 2022). Thus, the higher content of cyclopropane fatty acids in strain ATCH4^T^'s fatty acid profile might represent an adaptation to the harsh environmental conditions of the Atacama Desert.

**Table 3:** Cellular fatty-acid composition (%) of strain ATCH4^T^, 2, *I. loihiensis* L2-TR^T^ (data from Donachie et al. 2003) 3, *I. abyssalis* KMM 227^T^ (data from Ivanova et al. 2000); 4 *I. ramblicola* R22^T^ (data from Martínez-Cánovas et al. 2004)

Fatty acid	ATCH4^T^	*I. loihiensis*	*I. abyssalis*	*I. ramblicola*
C_10: 0_ 3-OH	–	–	–	1.1
C_11: 0_ iso	4.00	2	–	3.4
C_11: 0_ iso 3-OH	5.46	4.1	–	5.6
C_12: 0_ 3-OH	1.55	–	–	–
C_13: 0_ iso	2.17	1.8	1.0	1.5
C_13: 0_ iso 3-OH	5.25	3.3	–	2.3
C_15: 0_ anteiso	–	–	–	1.2
C_15: 1_ isoF	2.12	1.3	2.3	1.9
C_15: 1_ ω7c	–	–	1.3	–
C_15: 0_ iso	**23.84**	**32.6**	**33.7**	**24.7**
C_16: 1_ ω7c	-	6.0	7.0	5.2
C_16: 0_	7.26	7.6	6.3	7.4
C_16:0_ iso	1.3	–	–	–
C_16: 1_ ω7c	–	–	1.5	–
C_17: 1_ ω7c iso	–	**11.9**	-	**11.0**
C_17: 1_ ω8c	–	–	-	1.1
C_17:0_	1.17	–	-	1.7
C_17: 0_ iso	**17.69**	**11.0**	**11.9**	**12.9**
C_17: 0_ cyclo	**11.5**	1.7	-	2.5
C_18: 1_ ω7c	–	5.5	6.7	5.9
C_18: 1_ ω9c	–	1.0	1.4	1.2
C_18:0_	2.68	1.6	1.8	3.0
C_19:0_ cyclo ω7c	7.10	–	–	–

Values > 10% are indicated in bold writing

In accordance with previous reports, the main respiratory quinone found was ubiquinone 8 (Q8, 97.7%; Q7, 1.6%; Q9, 0.7%) (Song et al. 2013; Lee et al. 2015; Du et al. 2015).

### Taxonomic conclusions

Based on 16S rRNA gene sequence similarity scores, AAI, dDDH and ANI values, as well as various differentiating phenotypic and chemotaxonomic characteristics, strain ATCH4^T^ unambiguously represents a novel species of the genus *Idiomarina*, for which the designation *Idiomarina aminovorans* sp. nov. is proposed.

### Description of *Idiomarina aminovorans* sp. nov.

*Idiomarina aminovorans* (a.mi.no.vo’rans N.L. neut. n. *aminum* amine; L. pres. part. *vorans* devouring; N.L part. adj. aminovorans devouring amino acids).

Cells are Gram-stain-negative, strictly aerobic and motile by means of a monotrichous flagellum. They are slightly curved rods (1.5–3.5 × 0.5 µm). On IM4, colonies are circular, convex, smooth, beige, transparent, with irregular margins and 1–3 mm diameter after 3 days of incubation at 30°C. Growth occurs at 4–40°C (optimum 30°C), 3–12% (w/v) NaCl (optimum 7–10% (w/v)) and pH 6–9 (optimum 7.5–8). No growth was observed in the absence of NaCl. Cells tested positive for catalase and oxidase activities, H_2_S production and degradation of casein, pectin, gelatin and DNA. The cells are negative for amylase activity, as well as hydrolyzation of tween 40. Utilization of gelatin, pectin, glycyl-L-proline, L-alanine, L-arginine, L-aspartic acid, L-glutamic acid, L-histidine, L-serine, glucuronamide, p-hyroxy-phenylacetic acid, β-hydroxy-D,L-butyric acid, α-keto-butyric acid, acetoacetic acid, propionic acid and acetic acid as sole carbon source was observed. No growth was detected for dextrin, D-maltose, D-trehalose, D-cellobiose, D-gentiobiose, sucrose, D-turanose, stachyose, D-raffinose, α-D-lactose, D-melbiose, β-methyl-D-glucoside, D-salcine, N-acetyl-D-glucosamine, N-acetyl-β-D-mannosamine, N-acetyl-D-galatosamine, N-acetyl-neuraminic acid, α-D-glucose, D-mannose, D-fructose, D-galactose, 3-methyl glucose, D-fucose, L-fucose, L-rhamnose, inosine, D-sorbitol, D-mannitol, D-arabitol, myo-inositol, glycerol, D-glucose-6-PO_4_, D-fructose-6-PO_4_, D-aspartic acid, D-serine, D-galacturonic acid, L-galactonic acid lactone, D-gluconic acid, D-glucuronic acid, mucic acid, quinic acid, D-saccharic acid, methyl pyruvate, D-lactic acid methyl ester, L-lactic acid, citric acid, α-keto-glutaric acid, D-malic acid, L-malic acid, bromo-succinic acid, tween 40, γ-amino-butyric acid, α-hydroxy-butyric acid, and formic acid. The strain is resistant to fusidic acid, rifamycin SV, minocycline, vancomycin and aztreonam, but susceptible to troleandomycin, minocycline, lincomycin and nalidixic acid. Cells are positive for hydrolysis of gelatin and urea and negative for nitrate reduction, indole production, as well as hydrolysis of L-arginine, aesculin and p-nitro phenyl β-d-galactopyranoside (PNPG). Activity of alkaline phosphatase, esterase, esterase lipase, leucine arylamidase, cystine arylamidase, trypsin, acid phosphatase and naphthol-AS-BI-phosphohydrolase was noted. The main respiratory quinone is ubiquinone 8 (97.7%), while ubiquinones 7 and 9 are present at 1.6% and 0.7%, respectively. The predominant fatty acids are C_15:0_ iso (23.84%), C_17:0_ iso (17.69%) and C_17:0_ cyclo (11.5%).

The type strain is ATCH4^T^ (= DSM 114475 = LMG 32710), isolated from the surface water of the Llamará salt flat located in the Atacama Desert of northern Chile. The genomic G + C content of the type strain is 46.55 mol%. The GenBank accession numbers for the strain’s 16S rRNA gene sequence and draft genome are OM510467 and JAKNDA000000000, respectively.

## Supplementary Information

Below is the link to the electronic supplementary material.

Supplementary file1 (XLSX 17 KB)
